# Hairs in old books isotopically reconstruct the eating habits of early modern Japan

**DOI:** 10.1038/s41598-018-30617-0

**Published:** 2018-08-14

**Authors:** Atsushi Maruyama, Jun’ichiro Takemura, Hayato Sawada, Takaaki Kaneko, Yukihiro Kohmatsu, Atsushi Iriguchi

**Affiliations:** 1 Faculty of Science and Technology, Ryukoku University, Seta-oe, Otsu, Shiga 520-2194 Japan; 2 The Kinugasa Research Organization, Ritsumeikan University, Toji-in Kitamachi, Kita-ku, Kyoto 603-8577 Japan; 3 Ritsumeikan Global Innovation Research Organization, Ritsumeikan University, Noji-higashi, Kusatsu, Shiga 525-8577 Japan; 4 National Institute of Japanese Literature, Midori-cho, Tachikawa, Tokyo 190-0014 Japan

## Abstract

To complement literature-based historical knowledge of the eating habits of 17th- and 18th-century Japan, we analysed carbon and nitrogen isotope ratios (*δ*^13^C and *δ*^15^N, respectively) of human hairs embedded in cover paper of Japanese books printed during 1690s–1890s, taking regional and temporal variations into consideration. We purchased 24 book sets from second-hand book markets. Twenty-three sets contained enough human hairs, which were non-destructively extracted from the thick, recycled paper of the book covers and used to measure the *δ*^13^C and *δ*^15^N values, found to be identical within each book set. Relatively low *δ*^13^C values and high *δ*^15^N values suggested that people depended on rice, C_3_ vegetables, and fish, more exclusively than contemporary Japanese people. The relatively high *δ*^13^C values found in Edo (Tokyo) might be associated with the preference for C_4_ millets by Edo people as a measure against beriberi (locally recognised as the Edo affliction). The *δ*^15^N values gradually increased over 200 years, indicating an increase in the contribution of marine fish both as food and fertiliser for rice fields as suggested by literature-based studies. Further collection of hairs from books will enable a thorough examination of regional and temporal variations to better understand the pre-globalised food culture.

## Introduction

Reconstructing human eating habits from the early modern times (17th and 18th centuries) has both historical and cultural implications^1–6^. Generally, examining the regional and temporal variations of eating habits allows us to understand the transitions in social structure and cultural exchange. In the specific case of Japan, knowing the pre-westernisation or globalisation eating habits contributes to the understanding of food culture diversity at the basis of ‘washoku’ (traditional dietary cultures of the Japanese), an UNESCO’s intangible cultural heritage^3–6^. In addition, getting acquainted with the lifestyle in the largest cities of the early modern times (locally called the Edo period; 1603–1868 AD) is useful due to its high recycling and self-sufficiency rates^7,8^.

Past eating habits have been reconstructed from historical documents and illustrations^1–4,9,10^. Records on the lifestyle of Japanese ordinary people, including recipe books and diaries, increased since the early modern times^2^. However, special cases and events might be likely or unlikely to be recorded in historical documents. For example, records on prohibited and/or discriminated eating habits may be positively or negatively biased^9^. Thus, it is important to inspect and complement such literature-based knowledge with natural-scientific approaches that can reveal averaged, common and usual food habits. In ecological studies, the eating habits of animals are investigated using carbon and nitrogen stable isotope ratios (hereafter, *δ*^13^C and *δ*^15^N values, respectively), based on the constant difference in *δ*^13^C and *δ*^15^N values found in the diet and in animal tissues, designated as trophic discrimination factor (hereafter, TDF)^11–15^. Isotope analysis has also been applied by numerous studies to the reconstruction of past human eating habits, mostly using collagen extracted from skeletons^16–20^, which are neither abundant nor easily accessible. On the other hand, hair isotope ratios have typically been used to examine the regional variation of contemporary people’s eating habits, since hair collection is technically and ethically easy and hair-specific TDF is already known^21–25^. Consequently, if hairs of the past people can be collected with records on time and places, their eating habits can be accurately estimated and compared to those of contemporary times with the same TDF. Previous studies showed that amino-acid compositions of millennia-old hairs are well preserved and documented the diet in ancient human populations through isotope analysis of mummy hairs^26,27^.

**Figure 1:**
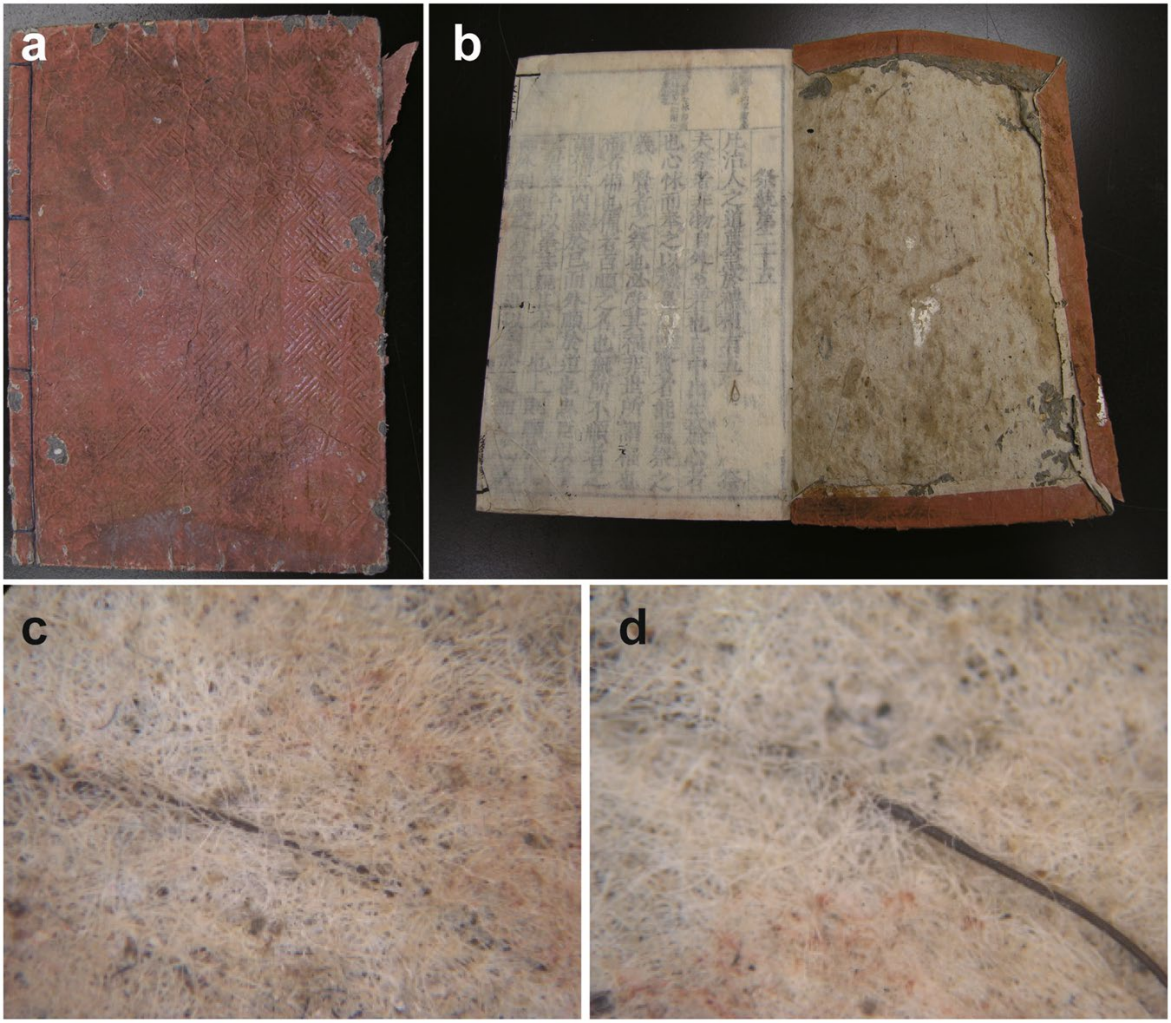
Photographs of an old book printed in the early modern Japan with human hairs. (**a**) External appearance of a book. (**b**) The recycled thick paper used for the cover (right side), where human hairs were embedded. (**c,d**) Human hairs embedded in the recycled thick paper of the book cover.

In this study, we focused on the fact that the recycled thick paper used for the covers of old books published in the early modern Japan often contains fragments of human hairs (Fig. 1). Book publication in Japan increased explosively and popularised since the middle of the 17^th^ century, reaching one million titles before the middle of 19^th^ century^28,29^. The covers of such books are made of recycled thick paper, which, for financial reasons, was believed to have been produced soon before book printing, using waste paper collected in the same cities where the books were printed. Because the hairs are embedded in the paper fibres, the hairs are thought to have been mixed accidentally during waste paper collection or blended intentionally for reinforcement during paper production. In either case, the hairs most likely belong to people living in the city and year of book printing, both of which are available from the records (colophon) on the book. Thus, the hairs found in each book, together with the records of time and place, constitute the ideal human tissue samples to reconstruct the eating habits at the time and place of the book printing, using isotope analysis. As a first step, this study validated the used approach by examining the isotopic variations within and between different book sets. The isotope analysis approach was then employed to reconstruct the eating habits of common people in the early modern Japan, and compare them with those of contemporary Japan, examining the temporal and regional variations.

## Results

### Collected books and extracted hairs

The latest publication (copyright transfer) year recorded from the collected 24 sets of the books, ranged from 1696 to 1865 (Table 1). The estimated time elapsed between the latest publication year and printing ranged from 0 to 150 years. Thus, the books were considered to have been printed between the 1690s and the 1890s. Difference in the elapsed time estimated by the two experienced bibliographers (A.I. and T.K.) was within 30 years, except for the two book sets (ID 55118, ID1053505), which were excluded from the analysis of changes in the *δ*^13^C and *δ*^15^N values. They were printed in Edo (present Tokyo, 6 sets), Kyoto (12 sets), Osaka (5 sets) and Nagoya (1 set), respectively. The number of hair fragments per book set ranged from 0 to approximately 1,000. All hair fragments extracted from the books were identified as human hair under the microscope. The number of replications for isotope analysis ranged from 0 to 22 per book set, depending on the amount of hairs (a total length of 12 cm was required for each analysis). One book set (ID 55118) containing no hairs was excluded from the isotope analysis. The entire list of 130 hair samples can be found with raw and mean *δ* values as Supplementary Tables S1–S3.

**Table 1:** List of the collected old book sets, from which human hairs were extracted for isotope analysis.

Publication/Printing					Numbers		
ID	Latest year	Lag	Latest publisher	City	Volumes	Hairs	Analysis
unregistered	1696	<10	Kawakatsu, G.	Kyoto	1	30	2
100182758	1712	0	Masu-ya, G.	Edo/Tokyo	3	20	9
1039298	1694	20	unknown	Kyoto^*^	1	138	3
588	1718	10	Izumi-ya Yamaguchi, M.	Kyoto	5	35	12
32940	1685	50	unknown	Kyoto^*^	1	105	2
57103	^J^1753	10	Horino-ya, J.	Tokyo	1	190	1
1053505	1771	**<10**	Kawachi-ya, G.	Osaka	1	408	3
64755	^J^1803	<10	Suda	Kyoto	5	1000	15
6735	1791	30	Shojoke-in	Kyoto	2	303	6
1034351	1776	50	Kiku-ya, K.	Kyoto	1	173	3
420199	1832	<10	Tawara-ya, J.	Kyoto	1	78	3
1558232	1785	50	Kiku-ya, S.	Kyoto	1	830	3
5674	1838	<10	Kawachi-ya, H.	Osaka	1	740	2
39474	1838	<10	Suhara-ya, I.	Edo/Tokyo	1	6	1
1013429	1838	<10	Kyoei-sya, S.	Edo/Tokyo	1	73	3
490095	1839	0	Itami-ya, Z.	Osaka	1	84	3
55118	1690	**150**	Hase-dera	Edo/Tokyo	5	0	0
1070548	1843	<10	Izumi-ya, I.	Edo/Tokyo	1	49	3
26120	1854	<10	Mino-ya, S.	Nagoya	8	179	22
61698	1857	<10	Kohchi-ya, H.	Osaka	3	97	9
2631	1860	<10	Fujii, M.	Kyoto	3	338	9
1715033	1861	<10	Seisyuh-bou	Osaka	1	290	2
13376	1854	20	Maekawa, G.	Kyoto	1	18	1
557222	1865	30	Hishi-ya, M.	Kyoto	5	25	13

Isotope ratios of 130 hair samples extracted from these book sets are available as Supplementary Tables S1–S3.

ID: document identification number by the National Institute of Japanese Literature, with which information including author name, title, and publication year is available in Japanese language at its web site (http://base1.nijl.ac.jp/infolib/meta_pub/G0001401KTG). ^J^before Latest year: printing year was only available from the preface. Lag: estimated elapsed time (year) after publication until printing (book sets with bold figures had two estimates different from each other by >30 years). *after City: printing city was estimated. Number of volumes: number of book volumes from which human hairs were extracted. Number of hairs: number of fragmented human hairs (of any length) observed in the book. Number of analysis: replications in the isotope analysis.

### *δ*^13^C and *δ*^15^N values of hairs in the books published in the early modern Japan

**Figure 2:**
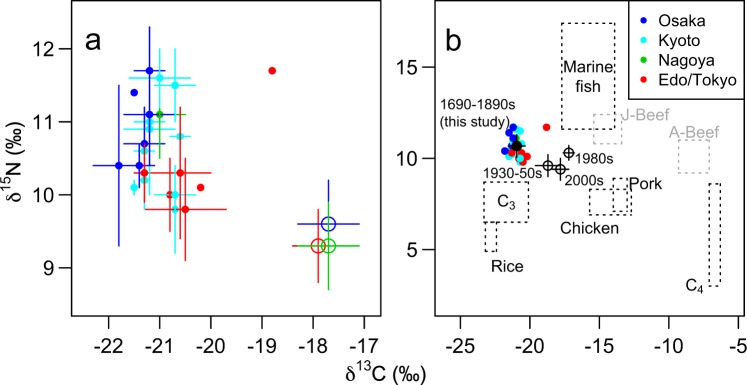
Scatter plots for the *δ*^13^C and *δ*^15^N values in human hairs and foods in Japan. Each solid coloured circle indicates mean *δ*^13^C and *δ*^15^N values of human hairs from the volumes that were concurrently printed with the same title (this study). Different colours indicate different printing cities. Bars are SD. (**a**) Comparison to the mean *δ*^13^C and *δ*^15^N values in the hairs of contemporary Japanese people in the corresponding regions (coloured open circles)^23^. (**b**) Comparison to the mean *δ*^13^C and *δ*^15^N values of human hairs in contemporary Japan (open black circles)^18,20,26^. Dotted squares indicate ranges of means + hair-specific TDFs (trophic discrimination factors; *δ*^13^C: +2.5‰, *δ*^15^N: +4.15‰)^21^ ± SD of the potential food sources^21,23,28,31^: rice, C_3_ vegetables (C_3_), C_4_ millets (C_4_), chicken, pork, Japanese beef (J-Beef), and American beef. Grey foods were rarely eaten in the early modern Japan. Modern and contemporary *δ*^13^C values are corrected for the Suess effect^51–54^; namely, 0.4‰, 1.0‰ and 1.6‰ were added to 1930–50s, 1980s and contemporary data, respectively.

No significant differences were found in the *δ*^13^C and *δ*^15^N values between the volumes in each of the 8 book sets, consisting of two or more volumes (analysis of variances (ANOVA), *p* > 0.05; Fig. 2a), except for the *δ*^13^C value of one book set (ID 61698) whose statistical result was within the range of false positives by multiple testing (ANOVA, *F*_2, 6_ = 7.09, *p* = 0.0263). On the other hand, significant differences were found among both the 8 book sets with two or more volumes (*δ*^13^C: *F*_7, 26_ = 5.066, *p* = 0.0010; *δ*^15^N: *F*_7, 26_ = 5.631, *p* = 0.0005) and the 23 book sets examined (*δ*^13^C: *F*_22, 26_ = 4.138, *p* = 0.0004; *δ*^15^N: *F*_22, 26_ = 2.837, *p* = 0.0060). Thus, the mean *δ*^13^C and *δ*^15^N values of hairs in each book set were used for further analysis.

The mean *δ*^13^C and *δ*^15^N values were not correlated (Spearman’s rank correlation coefficient, *r*_S_ = −0.0832, *p* > 0.5; Fig. 2), and were therefore treated as independent parameters. The means ± standard deviations of the mean *δ*^13^C and *δ*^15^N values within each book set were −20.9‰ ± 0.6‰ and 10.7‰ ± 0.6‰, respectively. On the scatter plot (Fig. 2b), these samples are plotted between TDF (*δ*^13^C: 2.5‰, *δ*^15^N: 4.15‰)-corrected values of marine fish and C_3_ vegetables^21,23,30^. When compared with the hairs of contemporary Japanese people^21,23,31^, our samples had distinctively lower *δ*^13^C values and slightly higher *δ*^15^N values.

The mean ± standard deviation of the mean *δ*^13^C and *δ*^15^N values within each book set were −21.4‰ ± 0.2‰ and 11.0‰ ± 0.5‰ in Osaka, −21.0‰ ± 0.3‰ and 10.7‰ ± 0.6‰ in Kyoto, −21.0‰ and 11.1‰ in Nagoya (*n* = 1), and −20.4‰ ± 0.9‰ and 10.4‰ ± 0.7‰ in Edo (present Tokyo), respectively. The mean *δ*^13^C value of hairs in each book set was different between printing places (ANOVA, *F*_2, 19_ = 6.529, *p* = 0.0070; Fig. 2a). *Post hoc* tests showed that the *δ*^13^C value was significantly higher in Edo than in Osaka (Tukey’s HSD, *p* = 0.0057). The difference in the *δ*^13^C value between Edo and Kyoto was marginal (*p* = 0.0526), whereas that between Osaka and Kyoto was not significant (*p* > 0.3). On the other hand, the *δ*^15^N value did not differ among printing places (*F*_2, 19_ = 1.338, *p* > 0.25).

**Figure 3:**
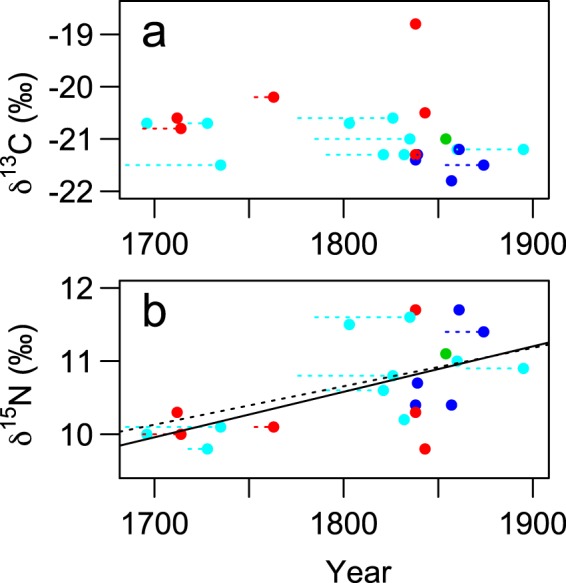
Relationship between the *δ*^13^C (**a**) and *δ*^15^N (**b**) values of human hairs in Japanese books printed during 1690s–1890s and printing year. Different colours indicate different printing cities (see legend of Fig. 2), which were pooled for the analysis. The horizontal dotted lines on the left of each plot indicate correction ranges based on the estimated elapsed time after publication until printing. Solid and dotted regression lines correspond to data after and before the correction, respectively. The Suess effect on the *δ*^13^C values^51–54^ is not corrected, because it is estimated less than 0.3‰ (≈ analytical errors) during this period.

The change in *δ*^13^C value of hairs in the books for approximately 200 years was not significant in either linear model (LM), before or after correction of the printing year with the estimated elapsed time since the latest publication year until printing (*p* > 0.25; Fig. 3). In contrast, the gradual increase in *δ*^15^N value was significant in both the before (*p* = 0.0125) and after correction (*p* = 0.0045).

## Discussion

Among the collected 24 book sets published in the early modern Japan, 23 contained a sufficient amount of hairs (>12 cm in total) for isotope analysis. All examined fragments were human, which is reasonable both in the case of accidental mixed-in and intentionally blending, since human hairs should have been most abundant in the cities. The finding that books published in the early modern Japan contained enough human hairs, strongly supports the use of isotopic approaches for reconstructing the eating habits of those days. Collecting hairs from books is considerably easier particularly when compared to the collection and treatment of collagen from skeletons, while collagen samples have a number of advantages over hair samples.

Unlike conventionally used collagen samples extracted from skeletons, hair samples from books may comprise a mixed sample from multiple individuals. Furthermore, bundled volumes that we assumed to have been printed concurrently and sympatrically might have been printed in different years or places. ANOVA results, however, showed that the variation in *δ*^13^C and *δ*^15^N values was negligible between volumes within the same book set, which supports that hairs from the same book set are isotopically identical. Thus, we concluded that *δ*^13^C and *δ*^15^N values of hairs can be analysed using the book set as a unit. These values are considered to represent the averaged eating habits of common people, living in the book printing city and year. Independency between the mean *δ*^13^C and *δ*^15^N values of hairs supports that the factors affecting isotopic variation can be independently explored.

On the scatter plot of *δ*^13^C and *δ*^15^N values, the hairs in the books published in the early modern Japan were plotted exactly between marine fish and C_3_ plants (vegetables and rice). The high contribution of rice corresponds to the literature-based knowledge that the production and consumption of rice increased drastically with the population explosion from 1600s to 1720s^32^. Importantly, the isotopic position cannot be occupied with any considerable contribution of the other foods including C_4_ millets, chicken, pork, beef, milk (mean *δ*^13^C + TDF: −17.1‰, mean *δ*^15^N + TDF: 9.3‰), cheese (−8.3‰, 9.9‰), ‘tofu’ (soybean curd; −23.7‰, 3.7‰) and ‘natto’ (fermented soybeans; −22.7‰, 4.7‰), according to the previous isotopic studies of contemporary foods in Japan^21,23,30,33^. It should be noted, however, that *δ*^13^C and *δ*^15^N values of foods depend on the production systems, which may be different between the early modern and contemporary Japan. In fact, a previous study reported lower *δ*^13^C values of skeletal collagen of a bird (raw *δ*^13^C: −20.8‰, raw *δ*^15^N: 11.2‰), a dog (−17.6‰, 11.6‰) and two pigs/boars (−19.4‰, 8.3‰; −21.9‰, 3.7‰) collected from Fushimi Castle site in Kyoto^18^. Contribution of freshwater fish is difficult to estimate, since the *δ*^13^C and *δ*^15^N values of freshwater fish are highly variable both temporally and spatially depending on eutrophication and other environmental conditions of the water bodies^34,35^ as well as species-specific trophic positions^36,37^. Thus, *δ*^13^C and *δ*^15^N values of hairs revealed that common people living in big cities of the early modern Japan depended on rice, vegetables and marine (and freshwater) fish, despite the variety of food menus present on the recipe books published in those days^3^. This conclusion corresponds to the results of isotopic studies using collagen samples extracted from human skeletons^18–20,38,39^. Previously-reported *δ*^13^C and *δ*^15^N values of only one hair sample from the book published in 1851 (−21.6‰ and 11.5‰, respectively^40^) are within the range of our samples, suggesting reproducibility of this method.

The comparison with the *δ*^13^C and *δ*^15^N values obtained from the hairs of contemporary Japanese people^23^ also supports that Japanese common people in the early modern times depended on foods with lower *δ*^13^C values, namely rice and C_3_ vegetables, than now. These results are consistent with historical facts whereupon, after the Meiji Restoration around 1860s, the Japanese eating habits were westernised, thereby people consumed more C_4_ corns and meats with higher *δ*^13^C values and lower *δ*^15^N values. In fact, prior to the Meiji Restoration, beef eating was prohibited and discriminated in Japan^9^. It also seems that regional variation was larger in the early modern times than now as suggested by an isotopic study using skeletal collagen^39^, although direct comparison is difficult since all our samples come from big cities whereas samples of contemporary people were widely collected, including the rural areas surrounding the corresponding cities^23^. The larger regional variation observed in the past might be due to limited food exchanges, compared to the contemporary Japan, where 62% (in 2016) of the whole foods are imported^23,41^.

Comparison within our samples showed that the *δ*^13^C values were higher in Edo (present Tokyo) than in Osaka and Kyoto, which implies that people in Edo depended more on foods with higher *δ*^13^C values than people in the other two cities. This regional difference may be due to Edo being the largest Japanese city, hence collecting a variety of agricultural and fisheries products^3^, some of which should have a higher *δ*^13^C value than rice, C_3_ vegetables and fish. A possible further explanation is that vitamin B1-rich C_4_ millets were preferred in Edo as a preventive measure against beriberi (a disease that affects the heart and circulatory system, caused by lack of vitamin B1), which used to be locally called ‘Edo wazurai’ (meaning the affliction of Edo) because the early modern people were affected by this disease only when they were visiting Edo, where vitamin B1-poor polished rice was most exclusively consumed^42,43^. Such cultural differences might be associated with the regional variation in *δ*^13^C values of hairs in the early modern Japan. The regional variation of diet in the early modern Japan was also suggested by the *δ*^13^C and *δ*^15^N values of skeletal collagen collected widely from rural sites in Japan^39^. The skeletal collagen from rural areas had larger variation in the *δ*^13^C and *δ*^15^N values (−21–−10‰ and 7–21‰, respectively) than our hair samples collected in the biggest cities, suggesting that the homogenization of Japanese feeding habits may have started from the big cities in the early modern Japan.

The within-sample comparison also showed that the *δ*^15^N values gradually increased over 200 years. This trend can be explained by the expansion of fish eating among ordinary people, as widely known from literature-based studies^3^. Otherwise, the *δ*^15^N values of rice and C_3_ vegetables may have increased in response to the increasing use of marine fish-based fertiliser in the agricultural field. In fact, the use of marine fish, such as sardines and herrings, as fertiliser in the early modern Japan is also recorded in historical documents^44^. A previous study also suggested that fish fertilizers might have been used for paddy rice in the early modern Japan based on a high nitrogen and sulphur stable isotope ratios of a sample of the rice hulls discovered in the inner coffin of a high-status female, who died in 1732^38^. Further collection and analysis of hair samples will be useful to test such detailed hypothesis in the future.

In conclusion, we demonstrated that isotope analysis of the human hairs found in old books printed during 1690s–1890s  characterised the eating habits of those days and revealed its regional and temporal variations. In previous isotopic studies, past eating habits were investigated using collagen extracted from skeletons^16–20^. The use of skeletons is advantageous to compare the eating habits between past individuals, since skeletons retain personal information on biological traits. In fact, these studies clearly showed differences in eating habits between sexes and ages in the past populations. In contrast, hairs collected from books are not useful for comparisons between individuals, since the hairs may be a mixture of multiple individuals and personal information from the owners is rarely available. Instead, the book records comprise the primary advantage of using hair samples. In fact, the printing year estimated from the book records can function as sampling year to find a rough trend of the eating habits over the 200 years used in this study, even before expert correction. Uncertainty observed in the estimated time elapsed between the latest publication year and printing may be decreased to some extent by selecting books, which tend to be printed only for several years (as an example, ID61698 is a guidebook on actors in fashion). Another advantage pertains the technical and ethical easiness of hair collection from the book, which is not usually be regarded as destruction of cultural properties in contrast to the case of archaeological skeletons. Moreover, isotopic studies of contemporary people are usually done with hair samples because of the technical and ethical easiness of hair collection^21–25^; thus, direct comparison of the isotope ratios with the same tissue-specific TDF is possible when the hairs are used for the study of past people. This is important given that inter-tissue and inter-species variations in TDF were recently shown as more considerable than previously believed based on earlier studies^24,25,45–47^. Books published in the early modern Japan remain massively available^28,29^, which has the potential to be used as ideal samples for large data analyses to test numerous hypotheses on regional variations and temporal changes of the eating habits and cuisines in the early modern Japan^3–7^. Further collection and analysis of hair samples are needed to understand the diversity of eating habits prior to globalisation.

## Methods

### Determination of the place and time, in which human hairs were mixed into old books

Twenty-four book sets were purchased from second-hand book markets in Tokyo, Kyoto and Kobe cities (Japan) at 300–3,000 JPY per volume. Each book set consisted of 1–8 volumes (54 volumes in total) sold under the same title, and with the same author and publication year (1685–1865). The volumes were sold as a bundle, thus were assumed to have been printed concurrently as a series (Table 1). The detailed time and place at which the hairs were mixed in the paper of the covers of each book set were determined as follows, according to the records in the book.

The publication year is usually recorded on the colophon (imprint) page of either the last volume or all volumes of each book set. For two book sets that lacked colophon pages, we used the years written in the preface. Importantly, most books published in the early modern Japan were printed using woodblocks, since type printing degenerated in the middle of 17^th^ century^28,29^. Hence, for book sets with two or more colophon pages reflecting copyright (woodblock) transfers from the original publishers to others, the latest records (mostly on the later page) were used for the analysis. Furthermore, given that the woodblocks can be repeatedly used after the publication (theoretically for hundreds of years), there may be a considerable time lag between the publication (i.e. the latest woodblock transfer) and printing of the book; thus, experts usually classify the early modern books into the first, early, and late prints by visually investigating the cracks and scratches of printed characters that reflect the age of the woodblock. In this study, an experienced bibliographer (A.I.) estimated the time elapsed after the latest publication year until printing, using periods of decades to correct the printing year (Table 1). Another experienced bibliographer (T.K.) cross-checked the elapsed time without knowing the estimates by A.I., and found the difference between the two researchers were <30 years, except for the two book sets (ID 55118, ID1053505), which were excluded from the analysis of changes in the *δ*^13^C and *δ*^15^N values. Moreover, book covers can be replaced by the owners, thereby increasing the time lag between printing of the main parts and producing the recycled paper for the covers. The same author (A.I.) therefore compared the paper quality and age between the book cover and main part by eye, thus excluding from the analysis the books whose covers were replaced after printing.

We used the city of the publisher recorded on the latest colophon page as the place of printing. For book sets that have two or more publishers in different places on the latest colophon page, the last (left-end) publisher’s place was used, as it is known that the last publisher solely printed the books whereas the other publishers cooperated by selling the books in other regions^29^. We assumed that, since recycled paper was produced only because it was cheap in those days, waste paper was collected and recycled in the same city where the books were printed and bound for the financial reasons.

### Extraction and isotope analysis of human hairs in the old books

The book covers made of the recycled thick paper were covered with thin paper, which was detached after spraying a small amount of pure water. Hair fragments of 0.5–20 cm length buried in the recycled thick paper (not only sandwiched between sheets) can be observed by eye (Fig. 1). Hairs were non-destructively extracted with forceps by spraying a small amount of pure water around the hairs. To avoid contamination of hairs after book printing, hairs that were only loosely attached at the paper surface or sandwiched between paper sheets were excluded from the analysis. The books were then dried in a desiccator at room temperature and preserved. Extracted hairs were observed under the microscope (×200–400). According to an established method used in legal medicine and food hygiene management in Japan^48,49^, hairs with <35% thickness ratios of the medulla to the whole hair, straight shapes and unsplit tips (if available) were considered as human head hairs.

Hairs were cleaned by soaking them in a 2:1 mixture of methanol and chloroform for 2 h, as previously described^21,22^, to remove lipids and any other surface contaminants. Before and after soaking, hairs were rinsed in distilled water for 10 min. using an ultrasonic cleaner. The hairs were then dried at 60 °C for 12 h. Hair fragments of 0.55–0.65 mg (corresponding to total length of approximately 10–12 cm) were packed into a tin cup for isotope analysis, which was performed with a Delta V Advantage mass spectrometer (Thermo Fisher Scientific, Bremen, Germany) connected to a Flash EA 1112 elemental analyser (Thermo Fisher Scientific) through a Conflo IV interface (Thermo Fisher Scientific) at Ryukoku University, Japan. Alanine (*δ*^15^N, 1.6‰ ± 0.2‰; *δ*^13^C, −19.6‰ ± 0.2‰) and histidine (−7.6‰ ± 0.2‰; −10.7‰ ± 0.2‰) standards were interspersed among samples (>10% of the total analyses) as standard reference materials for calibration and quality control. The *δ*^13^C and *δ*^15^N values were expressed as *δX* = (*R*_sample_/*R*_standard_) − 1, where *X* is ^13^C or ^15^N; *R*_sample_ corresponds to the ^13^C/^12^C or the ^15^N/^14^N ratio of the measured samples; and *R*_standard_ is the ^13^C/^12^C of the Vienna Pee Dee Belemnite or the ^15^N/^14^N of atmospheric nitrogen. The analytical accuracy and errors in the *δ* values were less than ±0.3‰.

### Data analysis

The *δ*^13^C and *δ*^15^N values of hairs were compared between the volumes within each of the 8 book sets that consisted of multiple volumes as well as between all book sets, using ANOVA. The independency of the mean *δ*^13^C and *δ*^15^N values was tested using Spearman’s rank correlation coefficient. The mean *δ*^13^C and *δ*^15^N values of hairs in the book sets were compared between printing places using ANOVA. Nagoya was excluded from the analysis, as only one book set printed in that city was collected and examined. For significant differences, a Tukey’s HSD *post hoc* test was used. The changes in the mean *δ*^13^C and *δ*^15^N values of hairs in the book sets were examined using LM. For each *δ* value, we employed two models, one of which used the latest publication year as printing year, whereas the other used the printing year corrected with the estimated elapsed time after the latest publication year until printing, as an independent variable. All statistical tests were performed with R ver. 3.2.2 software^50^, and a level of p < 0.05 was considered as statistically significant.

## Electronic supplementary material

Dataset 1

## Data Availability

The datasets generated during this study are available as Supplementary Information.
